# Cone-beam computed tomography reconstruction for a commercial proton beam therapy system

**DOI:** 10.1016/j.phro.2025.100745

**Published:** 2025-03-13

**Authors:** Josh W.H. Lindsay, Simon J.P. Meara, Matthew Clarke, Matthew Lowe, David Lines, Marianne C. Aznar, Marcel van Herk

**Affiliations:** a Division of Cancer Sciences, The University of Manchester, Manchester, UK; b Department of Radiotherapy Related Research, The Christie NHS Foundation Trust, Manchester, UK; c Christie Medical Physics and Engineering, The Christie NHS Foundation Trust, Manchester, UK

**Keywords:** CBCT, Reconstruction, IGRT, PBT, Proton therapy

## Abstract

**Background & Purpose:**

Cone-beam computed tomography (CBCT) images are used in image-guided radiotherapy to track anatomical changes throughout treatment and to set up patients to ensure accurate delivery of therapeutic radiation at each treatment session. An offline method of CBCT reconstruction workflow, operating on 2D projection images and specific to the imaging system in question, is needed for many image optimisation studies. Here we present a methodology to reconstruct CBCT images from these data for a commercial proton beam therapy machine, accounting for the variation in exposure and beam hardening from filtration due to gantry rotation during CBCT acquisition.

**Materials & Methods:**

Projection data of solid water phantoms were acquired to model bow-tie filter motion and beam hardening effects. Projection data and system CBCT reconstructions of a Catphan504 phantom were acquired for validation of the method, as well as a retrospectively accessed patient image. The presented workflow was assessed against the clinical reconstructions using uniformity, signal-to-noise-ratio, and contrast-to-noise-ratio measured in the phantom images.

**Results:**

The offline workflow eliminated crescent artefacts due to variable exposure and beam hardening in phantom and patient images. Signal-to-noise and contrast-to-noise ratios were similar compared to system reconstructions, although with slight differences thought to be due to interplay effects in the bow-tie filter.

**Conclusion:**

A workflow was developed to emulate the CBCT reconstruction process for a commercial proton therapy machine, providing a useful tool for optimised acquisition parameters and novel reconstruction processes using this system.

## 1 Introduction

In radiotherapy, cone-beam computed tomography (CBCT) images are used to position patients in order to ensure accurate delivery of the treatment. The benefits of CBCT are well-documented, allowing for sub-millimetre precision in patient set-up, potentially resulting in smaller planning target volume (PTV) margins and reduced dose to organ at risk (OAR) [1], [2], [3], [4]. CBCT also proves to be beneficial for visualising anatomical changes throughout treatment: for example, weight loss and tumour shrinkage can be monitored and this data provides advice for re-planning [5]. While new imaging and reconstruction protocols can be investigated to an extent in phantom studies, they cannot fully account for patient-specific phenomena such as anatomical noise [6]. Hence, to assess new protocols in a “real-world” setting without exposing patients to additional CBCT scans, it can be useful to apply new protocols to already-acquired clinical patient data retrospectively. Projection data can generally be recovered from commercial CBCT systems along with the reconstructed CBCT image. However, the specific physical effects (such as exposure, beam hardening, scatter, and motion of the detector and filter) that are corrected for in the projection images will vary from vendor to vendor. Some vendors will output projection images with some or all corrections made while others will integrate this step into the online reconstruction system. When reconstructing from projection data, a workflow equivalent to that found on the clinical machines must be used, which is generally unpublished. Without this, reconstructed images may significantly differ in image metrics, quality, and artefacts, giving an unrealistic representation of resulting images, particularly important if investigating potential clinical protocols. Without vendor-supplied reconstruction tools, offline methods must be developed for this purpose.

We present here a method to correct CBCT projection images from a commercial proton beam therapy machine, addressing variable exposure, the relative motion of the detector and filters during the gantry rotation, and beam hardening due to filtration and the imaged object. These corrected images are then reconstructed with open-source software and compared to reconstructed CBCT images from the system for validation.

## 2 Materials & methods

### 2.1 Imaging setup & data

**Fig. 1:**
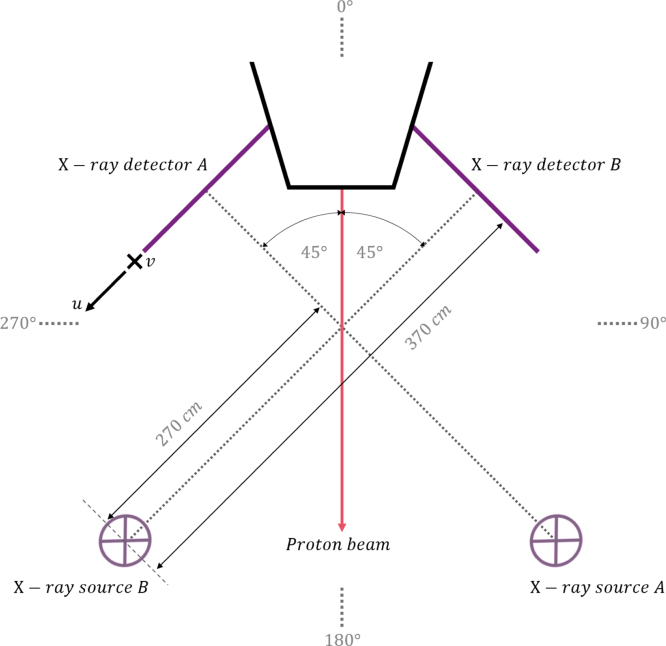
The ProBeam® system setup, showing the two detectors 90 ° from one another on either side of the treatment nozzle opposite their X-ray sources, in which the collimator, beam hardening filter, and BTFs are contained. In this illustration, the nozzle is shown at a position of 0 °, with sources A and B at 135 ° and 225 ° respectively. The detector coordinates relative to the gantry are also illustrated.

The ProBeam® (Varian Medical Systems, Palo Alto, USA) proton therapy machine is the subject of this work, a diagram of which is shown in Fig. 1; the CBCT imaging setup consists of two 39.7 x 29.8 cm flat panel detectors without anti-scatter grids affixed 90° apart on either side of the beam delivery nozzle. The detectors are 100 cm from the imaging isocentre and 370 cm from their respective kV X-ray sources, a larger distance than typical linac-mounted systems. The X-ray sources contain a collimator, beam hardening filter, and bow-tie filters (BTFs). Each source contains a full and half-fan BTF, each mounted on a rail that can automatically shift the appropriate filter into the position for full and half-fan acquisition modes. The purpose of a BTF is to produce a uniform exposure on the detectors by approximately complementing the attenuation of the imaged object. One source-detector pair at a time is used during CBCT acquisition, and a scan produces approximately 385 1024 x 768 pixel projection images in full-fan mode and approximately 695 in half-fan mode. Two sets of calibration projection images accompany this data by default; these are acquired as part of regular vendor quality assurance (QA) with all filters in place but without an object in field. Referred to as ‘air’ images, each set contains 10 equally spaced projections over a full 360 ° gantry rotation with one set acquired during clockwise rotation and one during counter-clockwise rotation. The acquired projection data and air images are automatically stamped with header data that includes gantry angle, X-ray source angle, and detector offsets along each plane. The lateral detector offset value records the total shift of the detector away from the gantry nozzle: the sum of sag-related offset and, if in half-fan mode, the intentional 16 cm offset for a larger field of view (FOV).

In this work, we aimed to emulate the clinical reconstruction of the ProBeam® system using both phantom and patient images. For this, 30 × 30x1 cm solid water phantoms were imaged to model beam hardening, and a Catphan 504 (The Phantom Laboratory, Greenwich, New York) was imaged for validation. All CBCT were acquired at 100 kVp full-fan mode at tube current of 154 mA, 15 ms exposure time per projection image. To avoid skinline truncation, exposure settings were such that even an air exposure would cause no saturation in the flat panel imager. Anonymised patient CBCT image and projection data acquired as part of routine treatment were used to validate our proposed reconstruction method. All patients consented to their data being used for research at the time of treatment and specific permission for this study was granted internally by the Proton Research Committee (PRC), (tracker number 2019-18).

### 2.2 Reconstruction process

The following method details the correction of a single projection image and is repeated for all 385 projections in a CBCT set. In subsequent notation, we refer to the angle for the projection image as ϕ, and the angles of the set of calibration air images for a particular rotation direction as $\theta_{j}$, where j=1,2,…,10 (denoting the 10 air images as described previously), each denoting the position of the active X-ray source at the acquisition of that image. Bold symbols denote 1024 x 768 image matrices and superscripts denote the corresponding angle of the X-ray source: $P^{\phi }$ is an acquired projection image at angle ϕ, and $A^{\theta_{j}}$ is an air image. A particular element in $P^{\phi }$ is written $P^{\phi }\left[u,v\right]$, where u and v are pixel coordinates on the detector. For a patient positioned head first, v is in the superior-inferior direction along the rotation axis of the gantry, and u is perpendicular to this.

**Fig. 2:**
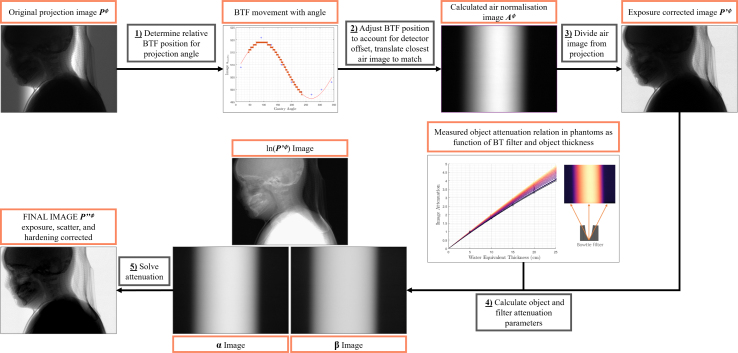
Flowchart summarising the projection image preprocessing method: (1) The position of the BTF is predicted using sample air calibration images; (2) An intermediate air image is created by shifting a calibration image according to the BTF and detector offsets; (3) The intermediate image is divided from the projection; (4) Free parameters of an experimentally determined effective attenuation relation are calculated; (5) The hardening equation is solved to give the corrected object attenuation.

The correction process for a single projection image is outlined in Fig. 2 and requires: (1) Modelling of the BTF position; (2) Exposure correction; (3) Estimation of hardening parameters; and (4) Correction for hardening in the image. These corrections were applied to the projection data using Matlab v. 2020b.

### 2.3 Prediction of BTF position

Relative motion of the filter, source, and detector due to gravity results in variable exposure across a projection image over an acquisition arc, which must be modelled and accounted for to avoid artefacts in the reconstructed CBCT image.

By assuming minimal rotation of the BTF during acquisition, and using recorded positional data of the detectors in each image header, the motion of the BTF can be decoupled from the detector and modelled using the set of air images $A^{\theta_{j}}$.

**Fig. 3:**
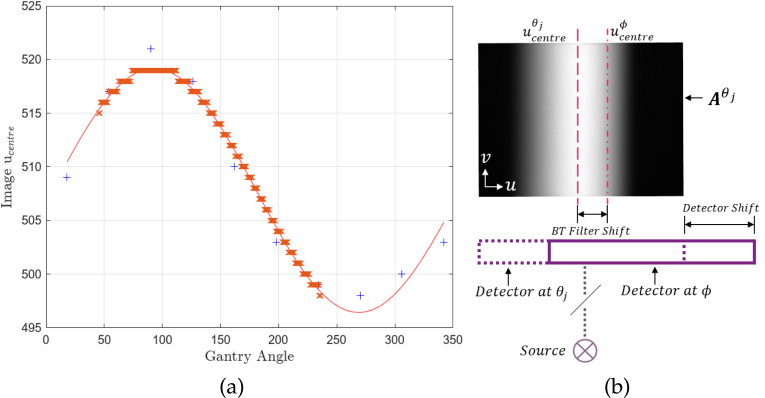
(a) Measured relative position of the BTF on the detector as a function of gantry angle. The blue crosses are the calculated $u_{\text{centre}}^{\theta_{j}}$ for each $A^{\theta_{j}}$ and the red line shows the fit C(θ) determined by least squares regression. The red crosses are evaluations of C(ϕ) for a set of 385 projection images, showing the predicted relative position to the nearest pixel of the BTF at each projection angle. (b) Illustration of how the separate shifts due to both the BTF and the detector during CBCT acquisition contribute to a change in exposure over the detector.

A reference column in each $A^{\theta_{j}}$, $u_{\text{centre}}^{\theta_{j}}$, is first estimated by:

(1) $$\overset{u_{\text{centre}}^{\theta_{j}}+L^{\theta_{j}}}{\underset{u=1}{\sum }}\overset{768}{\underset{v=1}{\sum }}A^{\theta_{j}}\left[u,v\right]=\frac{1}{2}\left(\overset{1024}{\underset{u=1}{\sum }}\overset{768}{\underset{v=1}{\sum }}A^{\theta_{j}}\left[u,v\right]\right),$$

where $u_{\text{centre}}^{\theta_{j}}$ is the column of pixels where the sum intensity up to that point is half of the total intensity of the image. $L^{\theta_{j}}$ is the lateral offset of the detector at $\theta_{j}$, determined from the header information in $A^{\theta_{j}}$. This reference column definition arises as the BTF shape varies only along u. $u_{\text{centre}}^{\theta_{j}}$ is a useful surrogate to measure the relative position of the filter independent of the detector and, in the case of a full-fan acquisition with a symmetric BTF, roughly aligns with the projection of the centre of the BTF. In half-fan mode, this reference point could still be used to track BTF motion although the reference column would not necessarily be at the centre point of the BTF. The relationship between $u_{\text{centre}}$ and θ is approximated by a sinusoidal curve, C(θ), an example of which is shown in Fig. 3(a).

C(ϕ) is then evaluated, giving the predicted relative position of the BTF at the angle of the projection image $P^{\phi }$. An air image whose acquisition angle is closest to the projection angle is selected, minimising $\left|\theta_{j}-\phi \right|$, and is shifted along its u axis such that $u_{centre}^{\phi }=C\left(\phi \right)$. Another shift is applied along u to account for detector shift utilising offset data from the header of $P^{\phi }$. Empty pixels are then filled in by their nearest neighbour and the whole image is normalised by the maximum intensity of $A^{\theta_{j}}$. This new air image, $A^{\phi }$, which is now specific to the projection acquisition angle ϕ and has accounted for filter and detector shifts, is then used to normalise exposure by:

(2) $${P{}^{\prime }}^{\phi }=P^{\phi }⊘A^{\phi }.$$

where ⊘ denotes Hadamard (elementwise) division of the two image matrices, and ${P{}^{\prime }}^{\phi }$ is the projection image corrected for variable exposure.

### 2.4 Correction for beam hardening

The Beer–Lambert law describes the attenuation of a monoenergetic X-ray beam of initial intensity $I_{0}$ as it traverses a homogeneous object of thickness x, resulting in the transmitted intensity I(x):

(3) $$I\left(x\right)=I_{0}\exp\left(-\mu \left(E,Z\right)x\right),$$

where μ(E,Z) is the object’s linear attenuation coefficient, which is a function of the X-ray energy E and material’s atomic number Z. However, CBCT imaging beams are polyenergetic. The measured intensity at the detector is therefore influenced by the specific spectrum, N(E), and the detector’s energy response, S(E), giving a modified Beer–Lambert law:

(4) $$I\left(x\right)\propto \int S\left(E\right)exp\left\{-\mu \left(E,Z\right)x\right\}N\left(E\right)dE,$$

describing a nonlinear relationship between object thickness and resulting X-ray intensity. In this work the Feldkamp-Davis-Kress (FDK) CBCT reconstruction algorithm has been used for reconstruction. However, this algorithm assumes a monoenergetic X-ray beam and so the intensities in projection images resulting from a polyenergetic beam must be linearised in line with Eq. (3).

Practically, S(E) and N(E) are not known. To circumvent this, the effective attenuation coefficient can be defined:

(5) $$-\mu_{\text{eff}}\left(x\right)x=\ln\left(\frac{I\left(x\right)}{I_{0}}\right),$$

representing the resultant “seen” attenuation averaged over a beam path. $\mu_{\text{eff}}$ can be determined experimentally by analysing image intensities in objects of known thicknesses [7].

As the BTF hardens the imaging beam prior to traversing the object, $\mu_{\text{eff}}$ also varies with the BTF thickness, i.e across elements of the projection images. An element of the exposure corrected projection image is then characterised as

(6) $$P^{\prime \phi }\left[u,v\right]=I_{0}exp\left\{-\mu_{\text{eff}}\left[u,v\right]X^{\phi }\left[u,v\right]\right\},$$

where $X^{\phi }\left[u,v\right]$ is the seen thickness of the imaged object at the element u,v at this angle.

$\mu_{\text{eff}}$ can be approximated by

(7) $$\mu_{\text{eff}}\left(x\right)=\alpha -\beta x,$$

where α and β are free parameters to be fitted from measured attenuation data. Since the seen thickness of BTF affects $\mu_{\text{eff}}$, we describe α and β in terms of the pixel intensities of the air image, as a surrogate for the BTF thickness. Eqs. (6), (7) can then be used to write

(8) $$P^{\prime \phi }\left[u,v\right]=I_{0}exp\left\{-\alpha \left(A^{\phi }\left[u,v\right]\right)X^{\phi }\left[u,v\right]+\beta \left(A^{\phi }\left[u,v\right]\right)X^{\phi }{\left[u,v\right]}^{2}\right\}.$$

The choice of fit in Eq. (7) gives a quadratic equation which can be analytically solved for the object thickness $X^{\phi }$ if the parameters α and β are calculated from $A^{\phi }$:

(9) $$\alpha \left(A^{\phi }\left[u,v\right]\right)X^{\phi }\left[u,v\right]-\beta \left(A^{\phi }\left[u,v\right]\right)X^{\phi }{\left[u,v\right]}^{2}+\ln\left(\frac{P^{\prime \phi }\left[u,v\right]}{I_{0}}\right)=0.$$

This can then be recomputed to attenuation values,

(10) $$P^{\prime \prime \phi }=I_{0}exp\left(-X^{\phi }\right),$$

giving the projection image $P^{\prime \prime \phi }$ for which both exposure and beam hardening have been accounted.

To model $\mu_{\text{eff}}$, CBCT images of 30 × 30x1 cm solid water phantoms were acquired at imaging isocentre, the setup for which is shown in Figure S1. Multiple slabs were stacked in order to image total thicknesses of 5, 10, 15, 20, and 25 cm. At source angle 0 ° (i.e phantom parallel to the detector), projections of these phantoms encompassed the whole FOV. These projection images, $W_{T}^{0}$, where T is the imaged thicknesses, were corrected for exposure by the method described in Section 2.3, and had their attenuation corrected for beam divergence so that each pixel in a particular $W_{T}^{0}$ described the intensity after transmission through T.

**Fig. 4:**
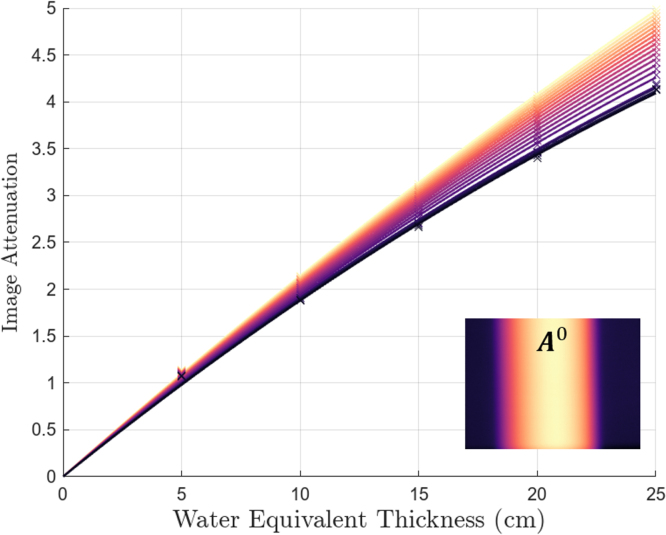
Plot of measured attenuation, $-\ln\left(W_{T}^{0}\right)$, as a function of water equivalent thickness T as measured from solid water phantom projection data. Here, elements in each projection image are binned according to the radiological length in $A^{0}$ at that element, shown in light to dark for smallest to greatest. Each function is fitted according to Eq. (7). The corresponding BTF attenuation image is shown inset. In the appendix, Figure S2 shows the fitted parameters for each of these curves as a function of $-\ln\left(A^{0}\right)$.

Each element of the water projection images was binned according to the corresponding value in $A^{0}$, essentially binning each pixel according to the seen thickness of BTF traversed at that point, resulting in Fig. 4. By comparison to Eqs. (5) and (7), the quadratic functions fitted to each set of binned values correspond to $\mu_{\text{eff}}$, and hence give the α and β for the particular filter thickness in that bin.

For each $P\prime \prime \phi_{i}$, Eq. (9) can be solved using the corresponding $A^{\phi }$ and the fitted functions in Fig. 4, allowing our final corrected projection image in Eq. (10) to be determined.

### 2.5 Reconstruction

After the corrections are applied, each $P\prime \prime \phi_{i}$ is downsampled by a factor of 2 in each dimension. The FDK algorithm with a Ram-Lak filter is then applied to these corrected projection images with *The Reconstruction Toolkit* (RTK) [8] version 2.1.0. The reconstructed volume is cropped to remove data outside of the FOV. The RTK reconstructions have cubic voxels, the size of which we set to 0.57 mm to match the pixel size in the ProBeam® CBCT images. The resulting images are then averaged over every four axial slices, to approximately match the slice thickness of 2 mm used in the clinical images.

### 2.6 Image evaluation

Regions of interest were identified in the Catphan and used to assess quantitative similarity between the offline and clinical CBCT. The homogenous region of the phantom was used to assess signal-to-noise ratio (SNR) and uniformity, and a number of inserts were used to assess contrast-to-noise ratio (CNR) at high, medium, and low contrast levels. Slice thickness and resolution were evaluated with the relevant phantom modules. A patient CBCT image was used to illustrate the visual impact of each processing step on artefact reduction, and for visual comparison of the CBCT from this method to clinical CBCT.

## 3 Results

**Fig. 5:**
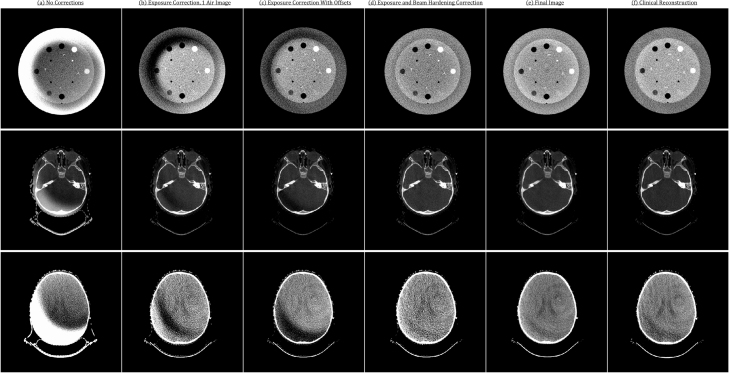
CBCT phantom and patient reconstructions with RTK with various pre- and post-processing corrections: (a) without any corrections; (b) exposure correction with a single air image; (c) exposure correction with filter and detector movement accounted for; (d) with filter movement and beam hardening correction; (e) all corrections with averaged slices after backprojection; (f) clinical reconstruction. Two positions are shown for the patient reconstructions to highlight bone and soft tissue contrast. A clear improvement and elimination of artefacts can be seen with each subsequent step.

Fig. 5 shows reconstructed images from RTK with each subsequent processing step applied. The reconstructions without any modifications to the projection data in (a) show dramatic cylindrical shading artefacts arising from a lack of exposure correction from the BTF. (b) shows exposure correction where one air image was used to normalise all projection images, resulting in a harsh crescent artefact due to the motion of the BTF during acquisition. In (c) these crescent artefacts are eliminated by shifting the air image, with slight shading remaining due to beam hardening. In (d) this shading is largely removed due to beam hardening correction, and (e) shows improved contrast and signal-to-noise ratio due to slice averaging.

Table 1 summarises image QA metrics measured in CBCT resulting from the offline method and those from the clinical system, as well as accompanying vendor-quoted nominal values taken from a provided user guide. SNR and Uniformity were in good agreement with the clinical scans and within the nominal range. Compared to clinical scans, high-contrast CNR (Teflon) was slightly higher in the offline reconstruction, and lower for medium-contrast (LDPE) and low-contrast (Acrylic) CNR. Figure S3 shows the slices used to evaluate CNR, slice thickness, and resolution.

**Table 1:** Table of typical QA metrics for the Catphan CBCT images using both the offline and clinical reconstruction methods. Uniformity is defined as the maximum deviation in average HU measured in four test ROI at the periphery of the uniform module of the Catphan, as compared to that in the centre. Vendor-quoted nominal values are shown for comparison.

QA metric	Offline	Clinical	Nominal
SNR	34.2	33.6	30–40
CNR (Teflon)	27.5	24.5	24.8
CNR (LDPE)	7.7	10.8	4.6
CNR (Acrylic)	0.74	1.51	1.8
Uniformity	18	14	<50
Resolution	6 lp/cm	7 lp/cm	>6 lp/cm
Slice thickness	2.0 mm	2.0 mm	2.0 mm

## 4 Discussion

This work presents a generalised solution to offline CBCT image reconstruction for a commercial proton therapy system without vendor-supplied software using minimal extra data. The workflow emulates the clinical reconstruction for CBCT images, with excellent visual agreement to clinically-acquired images (Fig. 5). Typical imaging metrics (signal-to-noise ratio, resolution, and uniformity) were within bounds that would be acceptable during routine QA checks. The differences in CNR between clinical CBCT and images from this method could be attributed to the lack of full deconvolution of object and filter effects which contribute to both scatter and beam hardening. Other analytical methods address this through imaging objects of varied thickness, both with and without filtration in place, in order to separate the object and filter contributions to scatter and hardening [9]. Due to vendor interlocks preventing full custom acquisition modes, unfiltered CBCT images could not be acquired and these methods could not be applied. If unfiltered imaging was made possible, this method could be easily modified and create images even closer to the clinical standard. Particularly in other body sites and patient sizes, more specific scatter correction may be required to achieve clinical-standard image quality. BTF “wobble” artefacts have been previously documented, and the correction method presented here is similar to that presented by Zheng et al. [10] who showed that nearest-angle air images can reduce crescent artefacts due to BTF shifts acquired with Varian On-Board Imager™ for X-ray radiotherapy and proposed a method of interpolating air images for exposure correction. The method presented here is of course on a different imaging system and has the added benefit of utilising exported image data to reduce additional calibration scans. In modelling the “wobble” effect, the presented method assumed that filter motion was in one dimension only, neglecting shifts in other directions and rotations. In principle, filter shifts along v should have a minimal effect on image quality as the filters are uniform in this direction. Rotations of the BTF and shifts along the beam direction (important due to magnification) have been somewhat accounted for by using the nearest air calibration image to a particular projection angle, relying on the assumption that motion outside of the u direction is minimal within the 36° around each air projection. Andersen et al. [11] presented an *a priori* scatter correction algorithm for the same system, using planning CT data to improve CBCT image quality and, like in our presented method, utilised RTK for their CBCT reconstructions before implementing their scatter correction method.

Projection data can be modified offline prior to applying this method, then processed and reconstructed in a way that is representative of that found on the imaging systems — a crucial step in CBCT studies that seek to optimise existing acquisition modes. Millimetre-order shifts in the detector and submillimeter shifts in the BTFs lead to considerable artefacts in reconstructed images if not accounted for due to the large magnification in the system. The method described here accounts for these minute variations in the on-board imaging system during CBCT acquisition. Within this imaging system, the calibration and correction for one source-detector pair cannot be applied entirely to another due to subtle differences in filter motion and beam spectra. As calibration data is acquired periodically, unique corrections can be generated for any set of CBCT projection images. For full precision, hardening models should be created for each source-detector pair using the method described, although slight differences in beam spectra have less of an effect on reconstructed images than the differences in filter motion. In other imaging systems, the method of filter shift calculation could also be applied, although the requirement for sensitive corrections is less obvious in smaller geometries where the same physical shift would only result in a small change on the detector due to smaller magnification. Such systems also have a relatively lower magnitude of focal spot blurring compared to those with larger geometries. Separating the detector and object effects and applying elementwise correction could still have use in small geometries, however, specifically in acquisitions which utilise filters with a large difference in attenuation across the field of view.

Although phantoms can be used to test custom acquisition modes, and some CBCT quality-enhancing algorithms operate on the reconstructed image, the use of a clinical reconstruction emulator is key in order to obtain a realistic representation of CBCT images that would result from simulation methods that operate in the projection domain. Modified acquisition parameters, iterative reconstruction algorithms, machine learning, or dual-energy based artefact reduction algorithms require offline reconstruction methods to allow for testing without taking extra patient images. By utilising the data provided by default in the clinical database, requiring minimal extra calibration images, and using open-source software, this workflow can be applied by any end user of this onboard imaging system, providing a crucial step in the evaluation of novel CBCT methods and clinical CBCT optimisation.

## CRediT authorship contribution statement

**Josh W.H. Lindsay:** Methodology, Software, Formal analysis, Visualisation, Conceptualisation, Writing – original draft. **Simon J.P. Meara:** Supervision, Writing – review & editing, Conceptualisation. **Matthew Clarke:** Resources, Writing – review & editing, Conceptualisation. **Matthew Lowe:** Resources, Writing – review & editing, Conceptualisation. **David Lines:** Resources, Writing – review & editing, Conceptualisation. **Marianne C. Aznar:** Supervision, Conceptualisation, Writing – review & editing, Funding acquisition. **Marcel van Herk:** Supervision, Software, Conceptualisation, Writing – review & editing.

## Declaration of competing interest

The authors declare that they have no known competing financial interests or personal relationships that could have appeared to influence the work reported in this paper.

## Appendix A Supplementary data

MMC S1 Supplementary material detailing the setup for measuring attenuation, the specific paramaters of the attenuation model, and additional image comparison.
