# Supplementary material for: Cone-beam computed tomography reconstruction for a commercial proton beam therapy system
Source: Phys Imaging Radiat Oncol. 2025 Mar 13;34:100745. doi: 10.1016/j.phro.2025.100745 (PMC12005303; doi:10.1016/j.phro.2025.100745)
Supplement: MMC S1 — Supplementary material detailing the setup for measuring attenuation, the specific paramaters of the attenuation model, and additional image comparison. [file mmc1.pdf]

SUPPLEMENTARY MATERIAL

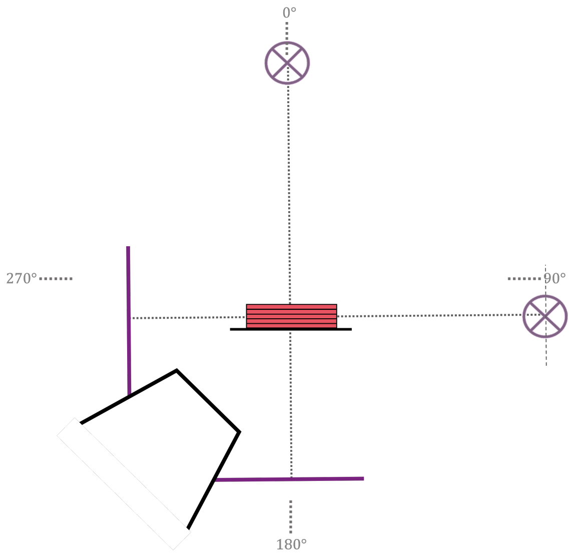

Supplementary Figure S1: Setup of the imaging system when scanning solid water phantom slabs to model μ_{eff}. Here, the detector is parallel with the face of the phantom at 0°, and the nozzle is positioned at 45°. This setup would be rotated 180° counter-clockwise to model for the other source-detector pair. In this positioning, the phantom covered the full FOV of the detector.

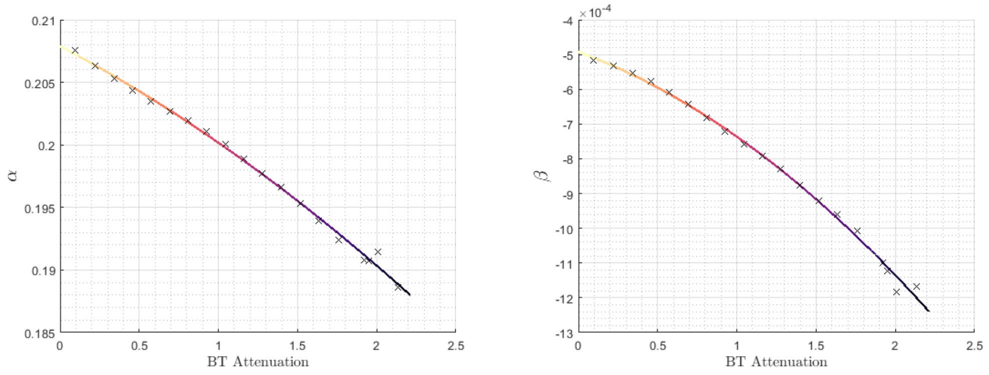

(a) (b)

Supplementary Figure S2: Fits for the free parameters in Equation **??** as a function of radiological length in $\boldsymbol{A}^0$, the black crosses are the parameter values in each bin of measured attenuation in $\boldsymbol{A}^0$, and fitted is a quadratic regression line coloured to reflect the bin value. These functions allow specific α and β maps to be calculated from a given air image.

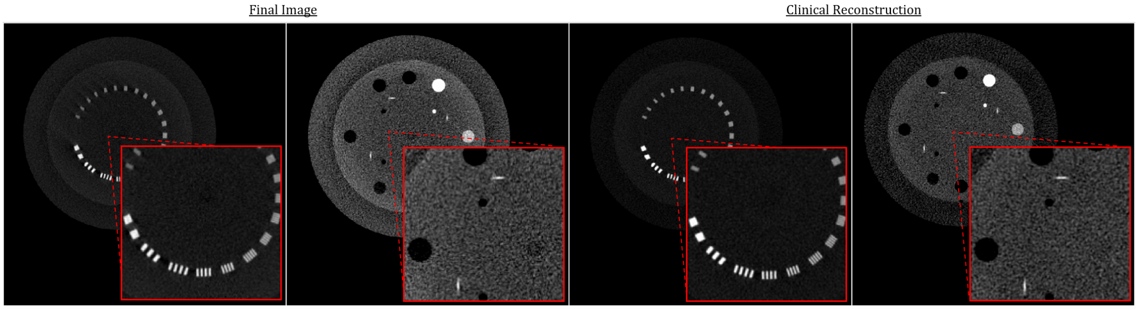

Supplementary Figure S3: Side-by-side of the final reconstructions of a Catphan 504 resulting from the presented workflow with clinical reconstruction for comparison. The resolution and slice thickness modules of the phantom are shown here, demonstrating the agreement of these parameters in each image. The resolution here is in line with the vendor nominal value of 6 l/cm and the slice thickness is approximately 2mm.

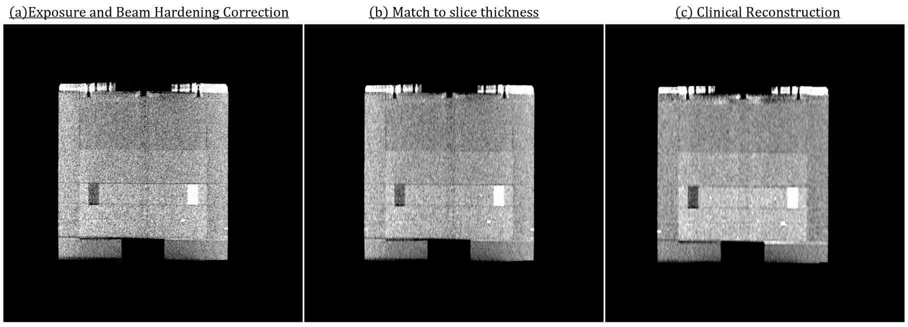

Supplementary Figure S4: Coronal view of Catphan reconstructions (a) without and (b) with slice averaging to match the (c) clinical CBCT image.

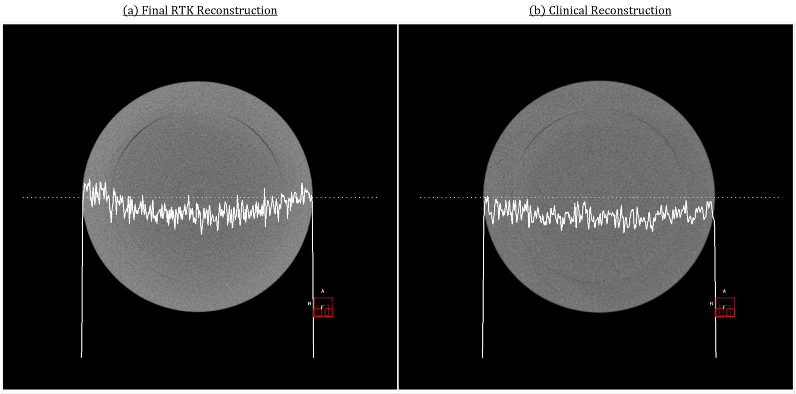

Supplementary Figure S5: Intensity profiles measured in the uniform module of the phantom in the (a) final reconstruction and (b) clinical CBCT image, 1100 HU level, 500 HU window.
